# Mouth Hygiene

**Published:** 1913-05-15

**Authors:** W. G. Ebersole

**Affiliations:** Sec'y-Treas., National Mouth Hygiene Association. 800 Scofield Bldg., Cleveland, Ohio


### MOUTH HYGIENE.

A SPECIAL FEATURE OF THE FOURTH INTERNATIONAL CONGRESS ON SCHOOL HYGIENE, INCLUDING PLANS OF ORGANIZATION AND CO-OPERATION.

BY W. G. EBERSOLE, M.D., D.D.S., SEC'Y-TREAS., NATIONAL MOUTH HYGIENE ASSOCIATION.

The dental profession of the world has been honored by an invitation from Dr. Thomas A. Storey, Secretary-General of the Fourth International Congress on School Hygiene, which meets in Buffalo, August 25—30, 1913, to participate in the most elaborate effort that has yet been made towards placing School Hygiene before the world in its true relation to the health, strength and working efficiency of the human race.

This is one of the most important opportunities that has come to the dental profession in its history for the purpose of presenting the various phases of Mouth Hygiene in their true relation to Hygiene in general.

At this meeting will be assenbled the largest number of people that have ever gathered in this country for the purpose of considering those questions which deal with School Hygiene. Not only the leading educators and school officials of this country, but of the world will be assembled on this occasion.

This means that every state in the Union will be represented by educational people and it is therefore highly important that every section of the country that is doing anything along the Mouth Hygiene line be represented in connection with the Scientific Exhibit dealing with the various phases of School Hygiene.

A large amount of space has been set aside to be devoted exclusively to the exhibition of material dealing with the various phases of Mouth Hygiene. It behooves every dentist that is interested in the Mouth Hygiene movement to see that his state, city or town has some sort of a display in connection with this work.

The organization of the Mouth Hygiene Literary and Scientific Exhibit part of the program has been placed in the charge of the writer of this article. An extensive literary program has been practically completed for that occasion.

That the Mouth Hygiene exhibit may be in keeping with the importance that Mouth Hygiene bears to School Hygiene in general, I am extending an invitation to the Oral Hygiene Committee of every dental organization in the country to participate in this exhibit. I am making an urgent appeal to each committee to see that its section of the country is represented by some sort of exhibit, setting forth either what they are doing or planning to do in that section.

Where a committee has nothing else to offer would suggest that they prepare a chart or a large card preferably black background with white lettering and framed in a black frame about an inch in width. The lettering on this card to be large enough to be read at a distance of twenty-five or thirty feet.

In organizing this work I am asking the Oral or Mouth Hygiene committees of the State Societies to assume responsibility for the State exhibit and requesting all other dental organizations to co-operate with the State committee in making a State exhibit but requesting that each individual committee present its exhibit as a component part of the State exhibit but retaining its distinctive feature. The State exhibits to become part of the National exhibit, each State becoming a component part of the National exhibit but retaining its individuality. The State exhibits will be arranged in alphabetical order so that the guests from any State will have no difficulty in ascertaining what is being done in that particular State.

The chairman of the various State organizations constitute a National committee, this committee to include the Oral Hygiene committee of the National Dental Association and the chairman of the various State committees appointed by the National Mouth Hygiene Association. The chairman of the National Dental Association's committee to be the executive officer of this National committee.

The National Mouth Hygiene Association has agreed to co-operate with the Congress to the extent of making its annual literary program a part of the Congress's literary program and is organizing its membership in the various States and cities along the same lines as suggested for the organized dental profession above.

In appointing its committee on exhibits it has followed out the policy of appointing those of its members who are known to be members of the State or Local Oral or Mouth Hygiene committees as its representatives. The National

Mouth Hygiene Association will also appoint one of its representatives to co-operate with Dr. Gram at Buffalo in arranging for exhibits.

We wish to call the attention of the profession to the fact that this is a tremendous undertaking on the part of the writer to organize this work along the lines suggested.

I do not have at my command the names of the committee-men of the various dental societies of the country and am therefore taking this means of notifying these committees of the part that they are expected to take in this work, requesting that they communicate with me at once indicating their willingness to co-operate and the style of exhibit that they expect to make.

I wish to say to the Oral Hygiene committees that if they are contemplating any work along the Mouth Hygiene lines they should have something in connection with the exhibit to indicate what they are doing or what the contemplate doing in order that the educational people from their sections of the country may find that they have a ive committe e in existence.

We would like to suggest to the State committees that they secure a large map of the State and indicate by means of various colored tacks the places and kinds of work that are being done.

I wish to impress upon the various State committees the importance of having their Mouth Hygiene exhibit a part of the National exhibit because of the fact that those in charge of this work expect to make a presentation of Mouth Hygiene part of the program which will be so impressive that every person who attends the Congress will be deeply interested in the Mouth Hygiene work. To have the exhibits split up and made a part of the States general hygiene exhibits would do much to lessen the impressiveness of the Mouth Hygiene exhibit.

W. G. EBERSOLE,
800 Scofield Bldg.,
Cleveland, Ohio.

